# The Persian Version of the Mobile Application Rating Scale (MARS-Fa): Translation and Validation Study

**DOI:** 10.2196/42225

**Published:** 2022-12-05

**Authors:** Saeed Barzegari, Ali Sharifi Kia, Marco Bardus, Stoyan R Stoyanov, Marjan GhaziSaeedi, Mouna Rafizadeh

**Affiliations:** 1 Department of Paramedicine Faculty of Paramedical Sciences Mazandaran University of Medical Sciences Sari Iran; 2 Department of Health Information Management School of Health Management and Information Science Iran University of Medical Sciences Tehran Iran; 3 Institute of Applied Health Research College of Medical and Dental Sciences University of Birmingham Edgbaston United Kingdom; 4 Creative Industries Faculty School of Design Queensland University of Technology Brisbane Australia; 5 Department of Health Information Management School of Allied Medical Sciences Tehran University of Medical Science Theran Iran

**Keywords:** mobile application rating scale, Farsi, mobile apps, validation, smartphone addiction, Persian, Iran, development, mobile health, mHealth, scale, validate, reliability, measurement tool, assessment tool

## Abstract

**Background:**

Approximately 110 million Farsi speakers worldwide have access to a growing mobile app market. Despite restrictions and international sanctions, Iran’s internal mobile health app market is growing, especially for Android-based apps. However, there is a need for guidelines for developing health apps that meet international quality standards. There are also no tools in Farsi that assess health app quality. Developers and researchers who operate in Farsi could benefit from such quality assessment tools to improve their outputs.

**Objective:**

This study aims to translate and culturally adapt the Mobile Application Rating Scale in Farsi (MARS-Fa). This study also evaluates the validity and reliability of the newly developed MARS-Fa tool.

**Methods:**

We used a well-established method to translate and back translate the MARS-Fa tool with a group of Iranian and international experts in Health Information Technology and Psychology. The final translated version of the tool was tested on a sample of 92 apps addressing smartphone addiction. Two trained reviewers completed an independent assessment of each app in Farsi and English. We reported reliability and construct validity estimates for the objective scales (engagement, functionality, aesthetics, and information quality). Reliability was based on the evaluation of intraclass correlation coefficients, Cronbach α and Spearman-Brown split-half reliability indicators (for internal consistency), as well as Pearson correlations for test-retest reliability. Construct validity included convergent and discriminant validity (through item-total correlations within the objective scales) and concurrent validity using Pearson correlations between the objective and subjective scores.

**Results:**

After completing the translation and cultural adaptation, the MARS-Fa tool was used to assess the selected apps for smartphone addiction. The MARS-Fa total scale showed good interrater reliability (intraclass correlation coefficient=0.83, 95% CI 0.74-0.89) and good internal consistency (Cronbach α=.84); Spearman-Brown split-half reliability for both raters was 0.79 to 0.93. The instrument showed excellent test-retest reliability (*r*=0.94). The correlations among the MARS-Fa subdomains and the total score were all significant and above *r*=0.40, suggesting good convergent and discriminant validity. The MARS-Fa was positively and significantly correlated with subjective quality (*r*=0.90, *P*<.001), and so were the objective subdomains of engagement (*r*=0.85, *P*<.001), information quality (*r*=0.80, *P*<.001), aesthetics (*r*=0.79, *P*<.001), and functionality (*r*=0.57, *P*<.001), indicating concurrent validity.

**Conclusions:**

The MARS-Fa is a reliable and valid instrument to assess mobile health apps. This instrument could be adopted by Farsi-speaking researchers and developers who want to evaluate the quality of mobile apps. While we tested the tool with a sample of apps addressing smartphone addiction, the MARS-Fa could assess other domains or issues since the Mobile App Rating Scale has been used to rate apps in different contexts and languages.

## Introduction

### Background

In the last 2 decades, advancements in mobile phone technology have allowed users the possibility to access health information from anywhere through nearly ubiquitous internet connectivity; at the same time, health care and public health organizations can diffuse health messages and provide diverse, continuous, indiscriminate support through mobile phones [1]. In July 2022, smartphones accounted for 4 in 5 mobile handsets available worldwide, with a global user base reaching 5.34 billion (an increase of 93 million since 2021), representing a penetration rate of 67% [2].

Undoubtedly, the public health and research communities consider mobile phones as preferred delivery modes in interventions addressing various health issues such as physical inactivity, substance misuse, and mental health [3-5]. Even a systematic review of mobile health (mHealth) interventions conducted in Iran showed that mobile phones (particularly SMS text messages) were increasingly used to deliver health interventions [6]. Some works argue that mobile apps include system design features that would prompt behavior change [7], with positive effects reported on physical function, pain intensity [8], physical activity [3], and mental health [9]. However, very little evidence exists on the sustained impacts of mobile apps on behaviors and health outcomes [3,4].

Nevertheless, the global mobile health app market does not seem to stop; it was valued at US $38.2 billion in 2021 and is expected to grow by nearly 12% between 2022 and 2030 [10]. According to Statista, the Iranian digital health market also follows a similar growth trend [11]. Some recent studies have highlighted the proliferation of mHealth in low- and middle-income countries such as Iran [12]. There are no official statistics about the number of smartphone users in Iran. Still, there are about 40 million active social media users, which could indicate technological adoption across the population [13]. With an estimated 150-220 million native speakers [14,15], mobile app development in the Persian language (or Farsi) seems particularly promising for local developers’ profitability. Industry-driven mHealth apps may offer a variety of advanced functions and capabilities. However, without the involvement of scientific expertise and evaluation, they may risk delivering unhelpful or potentially hazardous interventions [16-18].

Developers can easily leverage the limited application of guidelines in unregulated app markets that rely on open platforms such as Android in Iran, whose population has limited or no access to the global app markets on Google Play and Apple App Store. Two recently published reviews of the Iranian health app market identified about 3300 [19] and 3500 apps in the Android marketplace, which is the largest [20]. Two other reviews of COVID-19 apps in Iran searched for apps in different stores for iOS, such as CafeBazar, ParsHub, Charkhooneh, SibBazar, Sibche, SibApp, and SibIrani [21,22]. However, these stores are considered unsafe and unreliable by most Iranian citizens.

The proliferation of mobile health apps globally and in Iran raises concerns about their quality, accuracy, reliability, and efficacy [23]. According to some recent systematic reviews, various mHealth evaluation tools and rating scales have been developed to address this need [17,24]. These assessment tools vary from adapted website assessment tools to the use of consumers’ reviews or rating [25]. App store ratings are subjective and, by nature, a poor indicator of quality, medical usefulness, safety, or effectiveness. Quality reviews by trusted third parties can serve as landmarks in assessing the security, validity, and quality of mHealth apps [26]. According to a review of health app evaluation tools by BinDhim et al [25], the most frequently used were the Royal College of Physicians’ Health Informatics Unit Checklist [27], the Organization for the Review of Care and Health Applications-24 Question Assessment (ORCHA-24) [28], and the Mobile Application Rating Scale (MARS) [29]. The Royal College of Physicians’ Health Informatics Unit Checklist only looks at the developer, the functionality, and whether the app has been evaluated effectively in related interventions [27]. The Organization for the Review of Care and Health Applications-24 Question Assessment focuses on data governance, clinical impact and assurance, and user experience and engagement as quality aspects [28], but it fails to provide a comprehensive, multidimensional evaluation of app quality. Conversely, the MARS assesses app quality on a broader and more diverse range of criteria or domains, such as engagement, functionality, aesthetics, and information quality. According to Azad-Khanegah and colleagues [24], the MARS provides a multidimensional, reliable, and flexible app-quality rating scale for researchers, developers, and health care professionals [29]. The MARS has been used to evaluate apps in user-based heuristic evaluations [30] and expert-driven content analyses of apps [31-33]. The MARS has been validated across multiple studies [32] and translated into Italian [34], and more recently into German [35], Spanish [36], Arabic [37], Japanese [38], Korean [39], French [40], and Turkish [41]. However, this instrument has no translation or cultural adaptation for the Farsi language.

### Objectives

This study aimed to (1) translate and culturally adapt the MARS in the Farsi language (MARS-Fa) and (2) validate the tool by examining its psychometric properties.

## Methods

### Study Design

This study followed a 2-step process, starting with the translation and cultural adaptation of the MARS in English to Farsi, as done in the validation studies mentioned above [34-41]. The second step involved a statistical evaluation of the MARS-Fa’s reliability and validity.

### Original Instrument: The MARS

The MARS [29] consists of 29 items divided into the following 4 objective subscales: *engagement* (items 1-5), *functionality* (items 6-9), *aesthetics* (items 10-12), and *information* (items 13-19); it also comprises a subjective subscale, which is *app subjective quality* (items 20-23). The MARS also includes items intended to measure the *perceived impact of the app* for the intended end users. The perceived impact scale includes 6 additional items that evaluate the app’s potential to affect users’ knowledge, awareness, and intentions to perform the target behaviors. However, it is intended for the end users and is generally not used to assess app quality or to compare apps. All items are rated on 5-point scales, usually ranging from 1 (“poor”) to 5 (“excellent”), except for the *perceived impact* items, which are based on 5-point Likert-type scales, where 1 is “strongly disagree,” and 5 is “strongly agree.” According to the guidelines from the original MARS study [29], an average score is calculated for each subscale. A total app quality score represents the average of the 4 objective subscales. The original MARS study reported high internal consistency (Cronbach α=.90) and reliability, with intraclass correlation coefficients (ICCs) averaging 0.79 [29].

### Translation and Adaptation Process

Following the so-called “universalist approach” [42] applied in other MARS validation studies [37], the translation and adaptation process consisted of the following steps. First, a translation was conducted, including essential item and conceptual equivalences, which were evaluated and validated by a panel of 8 experts, including 4 PhD students in health information management and health information technology, and 4 researchers with PhDs in psychology and nursing. In the next step, 2 English translators familiar with IT concepts independently translated the MARS tool into Farsi. A semantic evaluation was also performed to check the ambiguity and simplicity of the Farsi translation among the potential target population. Finally, to ensure that the Farsi version was perceived as the original English scale, it was translated back to English by a bilingual translator and compared with the original version. The back-translated version was finally checked and validated by the developer of the original MARS [29], and a few amendments were made.

### Sample Selection for Scale Validation

To assess the reliability and validity of the MARS-Fa, we selected a sample of health apps targeting smartphone addiction and available on the Android and iOS stores. While the MARS is intended to address health apps of any domain, our study team included researchers with solid expertise in health information technology and smartphone addiction.

**Figure 1:**
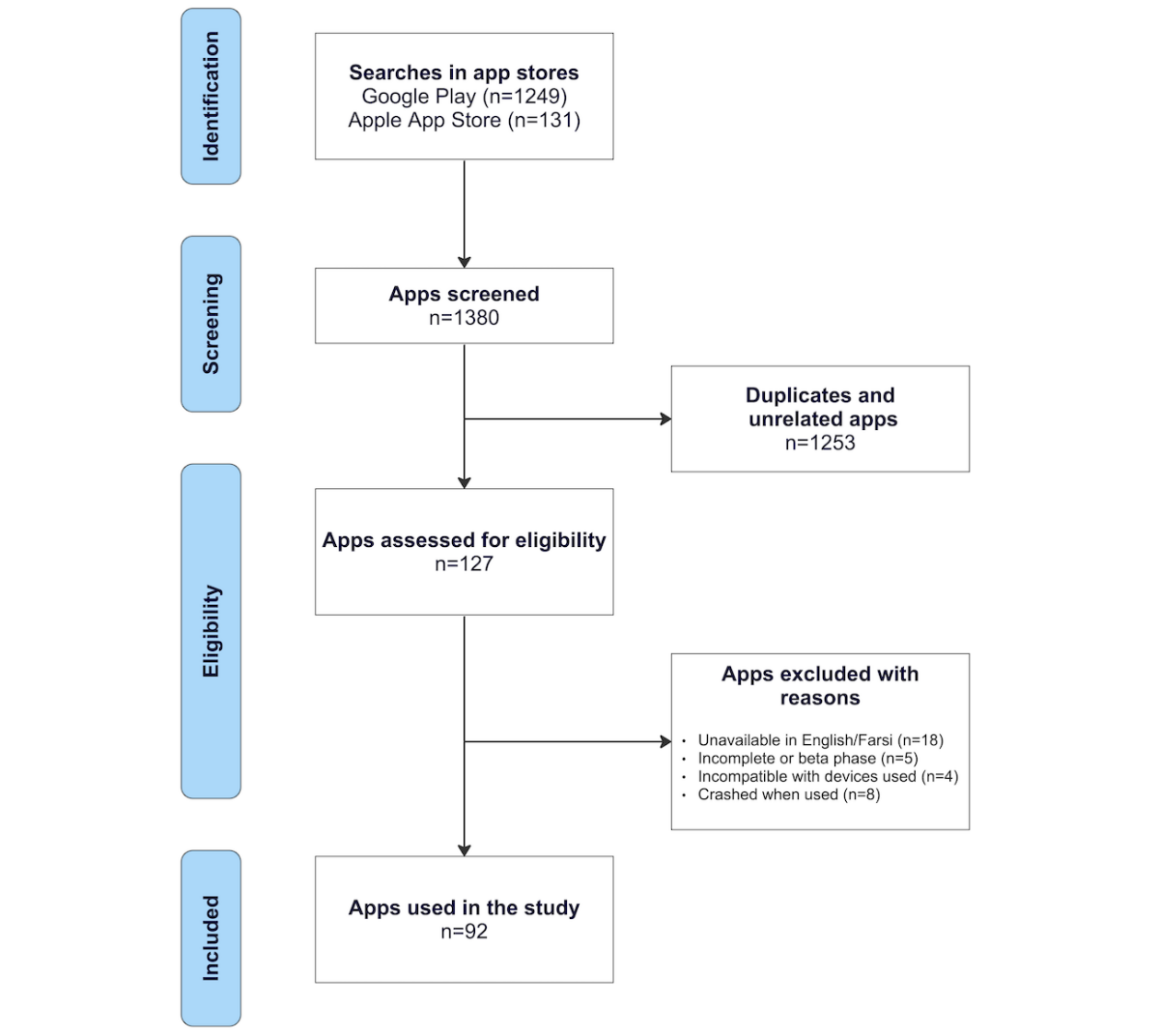
Flow diagram of the app selection process.

A systematic process was followed to select the smartphone addiction apps for evaluation. All steps are presented in the diagram in Figure 1. Two Health Information Technology experts independently searched the Google Play and Apple App stores on May 22 and June 1, 2019. The keywords included “Smartphone Addiction,” “Phone Addiction,” “Mobile Phone Addiction,” “Cellphone Addiction,” and “Nomophobia.” To be included in the sample, apps had to (1) be available in either English or Farsi languages, (2) address smartphone addiction, and (3) be free of charge. Exclusion criteria were as follows: (1) apps being underdevelopment or not released yet; (2) apps that were unavailable or that could not be downloaded due to device incompatibility; (3) apps failing to launch after 3 attempts or apps crashing. The app selection process is summarized in Figure 1.

### Validation Process

Two raters had a session to study the MARS tool and discuss their perception regarding its concepts. As a result, both raters came to a shared understanding of how to use the MARS for the app target group. Both raters downloaded each selected app on both iOS and Android-based smartphones. They completed an independent assessment of each app in both Farsi and English.

Initially, the 2 raters independently evaluated 10 apps for about 10 minutes each. The similarity between the reviewers’ judgments was assessed by comparing ICCs, as done in the original MARS study [29]. This step was introduced to establish a minimum interrater reliability level and allow the raters to identify and discuss differences and address inconsistencies before assessing the remaining apps. After 2 weeks, 10 apps were randomly selected and evaluated for the second time by the same 2 raters to evaluate their test-retest reliability.

In the next step, out of the selected 92 apps, 45 (49%) were randomly chosen for the validation exercise. This number was deemed sufficient to reach an empirical assurance of 90% and an assurance probability of .15, as done in the study that brought about the development of the Italian version of the MARS [34].

### Ethical Considerations

This study involved secondary analyses of research data without including human participants; as such, no ethical approval was needed.

### Statistical Analyses

Descriptive statistics were calculated for all items, subscales, and the total MARS scale, including means, standard deviations, and asymmetry coefficients. Subsequently, the reliability and validity measures of the MARS were evaluated separately for both raters. Interrater differences were assessed for subscale scores.

ICCs, using a mixed 2-factor model, were used to evaluate the interrater reliability [43]. This method has been deemed appropriate, as it accounts for the proximity of scores rather than an absolute agreement between raters. ICC values less than 0.50, between 0.50 and 0.75, between 0.75 and 0.90, and greater than 0.90 are respectively considered poor, moderate, good, and excellent interrater reliability [43]. Cronbach α was used to assess internal consistency and was interpreted as excellent (≥0.90), good (0.80-0.89), acceptable (0.70-0.79), questionable (0.60-0.69), poor (0.50-0.59), and unacceptable (<0.50), as reported in [30]. Split-half reliability was used to evaluate the internal consistency of the average of the 2 raters using the Spearman-Brown prophecy formula, as used in the Italian MARS validation study [34]. Pearson correlations were used to assess the test-retest reliability [43].

To determine construct and concurrent validity, we replicated the approach of Yamamoto et al [38], who validated the Japanese MARS. Construct validity was based on evaluating item-subscale correlations [38] for the objective scales only, considering the intrinsic subjectivity of the “subjective quality” scale. Convergent validity was deemed satisfactory when an item achieved a correlation above *r*=0.20 with the respective subscale, a threshold used in the Italian [34] and Japanese [38] validation studies. Discriminant validity was deemed satisfactory if more than 80% of the correlation coefficients were higher than those with other subscales [38]. To establish concurrent validity, we examined the correlations between the MARS-Fa objective scales and the subjective quality, given that there are no gold-standard app quality indicators other than the MARS itself [38]. Other studies have compared the MARS objective and subjective scores to the average app store ratings for each app [37,38]; however, these were deemed inappropriate as the Farsi version of the app pages include few reviews and ratings that might be biased and manipulated, hence being unreliable indicators of app quality.

## Results

### Translation and Adaptation Process

In the forward and backward translation and face validation phases, we used IT and health experts, who identified common words, phrases, and sentences in both disciplines. There were some corrections made after the backward-translated version was reviewed and edited by 2 authors (one of them was the corresponding author of the original scale). The final version of the translation was deemed clear and understandable for both groups and not in conflict with the original version. Table 1 shows the words that were corrected in the process. The final version of the MARS-Fa tool is available in Multimedia Appendix 1.

**Table 1:** Corrections on the back-translated Mobile Application Rating Scale (MARS, in English) and the Farsi version (MARS-Fa).

First Farsi translation	Retranslated word	Correct word	Corrected Farsi word
سرگرمی	Entertainment	Engagement	درگیر سازی
استفاده مداوم	Constant use	Frequent use	استفاده مکرر
ناسازگار	Incompatible	Incoherent	غیرمنسجم
محدودکننده	Overwhelming	Was explained as “too much for the user to know where to start”	منکوب کننده
واضح	Obvious	Intuitive	منطقی؛ مبنی بر درک و انتقال مستقیم

### App Selection Process

Initial searches in the app stores yielded 1380 apps from both Android and iOS stores. After removing duplicates and irrelevant apps (n=1253), 127 apps were screened for inclusion. Of these 127 apps, 18 (14.2%) were excluded because they were not available in either Farsi or English, 5 (3.9%) were excluded because they were incomplete (beta versions), 4 (3.1%) because they were incompatible with the devices used to test the apps, and 8 (6.3%) could not load (crashed when launching them), leaving a final set of 92 (72.4%) apps for the validation study (Figure 1). An ID was assigned to each app. In the next step, 45 apps (Multimedia Appendix 2) were randomly and with equal proportions selected from the two app stores (Google Play: n=30, 67%; App Store: n=15, 33%) for preliminary testing. The apps included in this study lacked any peer-reviewed publications of formal efficacy trials. Hence, item 19 of the information domain, “Evidence base,” which aims to assess the app’s reported efficacy based on randomized controlled trials, was excluded from the calculations as none of the apps were formally trialed.

### Reliability and Validity Analyses

Table 2 presents the descriptive statistics for each subscale and the total MARS-Fa score separately for each rater. As the responses followed a nonnormal distribution, nonparametric tests were used to check the differences between raters. The paired *t* test and the Wilcoxon test (2-tailed) showed no significant differences between the raters’ mean scores.

Table 3 reports the results of the reliability analyses. Interrater reliability was good for “engagement” (ICC=0.85), “information quality” (ICC=0.76), and “aesthetics” (ICC=0.75), and moderate for “functionality” (ICC=0.60). ICC was also good for the MARS-Fa total score (0.83) and “subjective quality” (0.78). The Spearman-Brown split-half reliability estimates ranged between 0.79 and 0.93, confirming good interrater reliability.

Cronbach α coefficients for each of the MARS-Fa subdomains, total, score, and subjective quality (Table 3) ranged from .51 to .89 for the first rater and .56 to .84 for the second rater. The average alpha coefficient was .84 for the total MARS-Fa and subjective quality. Spearman-Brown split-half reliability indicators were very good and excellent, ranging from 0.79 for functionality and impact and 0.93 for the MARS-Fa total score.

The MARS-Fa total score and subscales had excellent and good test-retest reliability, with correlations above 0.90, indicating no significant change over time (*P*>.05) for all objective subscales, total score, and subjective quality score. Overall, the average test-retest correlation between the 2 raters was high (*r*=0.94).

**Table 2:** Descriptive statistics and interrater comparisons.

Scale	Minimum-maximum			Skewness			Shapiro-Wilk (*P* value)			Mean (SD)			*P* value^a^		Cohen *d*
	R1^b^	R2	R1		R2	R1		R2	R1		R2				
Engagement	1.60-4.60	1.60-4.60	–0.40		–0.43	0.94 (.02)		0.97 (.40)	3.40 (0.87)		3.31 (0.69)	.11		0.24	
Functionality	2.00-4.75	2.25-4.50	–0.50		–0.96	0.96 (.14)		0.91 (<.001)	3.68 (0.57)		3.73 (0.54)	.47		0.11	
Aesthetics	2.33-5.00	2.33-5.00	–0.74		–0.46	0.92 (<.001)		0.93 (.01)	3.90 (0.64)		3.79 (0.74)	.16		0.21	
Information	1.75-4.50	1.75-4.17	–0.54		–0.28	0.96 (.14)		0.96 (.08)	3.28 (0.57)		3.31 (0.54)	.67		0.06	
MARS-Fa^c^ total score	2.17-4.66	2.48-4.50	–0.40		–0.29	0.97 (.30)		0.97 (.36)	3.57 (0.56)		3.54 (0.52)	.53		0.001	
Subjective quality	1.00-4.75	1.20-4.75	–0.52		–0.19	0.91 (<.001)		0.94 (.03)	3.32 (1.19)		3.24 (1.00)	.34		0.14	

^a^*P* value of the Wilcoxon W or *t* test.

^b^R: reviewer.

^c^MARS-Fa: Mobile Application Rating Scale in Farsi.

**Table 3:** Interrater reliability, internal consistency, and test-retest reliability results.

Scale	Cronbach α		ICC^a^ (95% CI)	Spearman-Brown split-half reliability	Test-retest reliability (Pearson *r*)	
	R1^b^	R2			R1	R2
Engagement	.89	.83	0.85 (0.72-0.90)	0.92	0.94	0.96
Functionality	.51	.56	0.60 (0.39-0.74)	0.79	0.93	0.96
Aesthetics	.71	.83	0.75 (0.62-0.84)	0.85	0.95	0.91
Information	.77	.65	0.76 (0.63-0.84)	0.86	0.92	0.89
MARS-Fa^c^ total score	.84	.84	0.83 (0.74-0.89)	0.93	0.92	0.95
Subjective quality	.84	.84	0.78 (0.67-0.86)	0.82	0.94	1.00

^a^ICC: intraclass correlation coefficient.

^b^R: reviewer.

^c^MARS-Fa: Mobile Application Rating Scale in Farsi.

### Construct Validity

The item-total correlations are shown in Table 4, all of which were above 0.40 in the objective subscales except for functionality item 7, “Ease of use” (*r*=0.27). Success rate was deemed satisfactory for convergent validity. Overall, success rate was also deemed satisfactory for divergent validity, with all items being above the threshold in all subdomains except functionality (item 7), and information quality (item 13, “Accuracy of app description”), which had the lowest correlation with the total among the other items of the domain.

Pearson correlations between the MARS-Fa total score, the respective objective subdomains, and the subjective quality score are shown in Table 5. The MARS-Fa was positively and significantly correlated with subjective quality (*r*=0.90, *P*<.001), and so were the objective subdomains of engagement (*r*=0.85, *P*<.001), information quality (*r*=0.80, *P*<.001), aesthetics (*r*=0.79, *P*<.001), and functionality (*r*=0.57, *P*<.001). The relationships between the MARS-Fa and the objective domains are not reported because the MARS-Fa is their composite score. The relationships among the objective domains were also significant (*P*<.001).

**Table 4:** Construct validity indicators.

MARS^a^ objective subscale items		Corrected item-total correlations (Pearson *r*)	Success rate			
			Convergent validity		Divergent validity	
**Engagement**				5/5		5/5
	Entertainment	0.72				
	Interest	0.70				
	Customization	0.64				
	Interactivity	0.74				
	Target group	0.83				
**Functionality**				3/4		3/4
	Performance	0.65				
	Ease of use	0.27				
	Navigation	0.50				
	Gestural design	0.38				
**Aesthetics**			3/3		3/3	
	Layout	0.70				
	Graphics	0.79				
	Visual appeal	0.81				
**Information**				6/6		5/6
	Accuracy of app description	0.43				
	Goals	0.67				
	Quality of information	0.57				
	Quantity of information	0.68				
	Visual information	0.51				
	Credibility	0.47				
	Evidence base	N/A^b^	N/A		N/A	

^a^MARS: Mobile Application Rating Scale.

^b^N/A: not applicable.

**Table 5:** Correlations between the Mobile Application Rating Scale in Farsi (MARS-Fa) objective scores and subjective quality.

Relationship	Pearson *r*	Lower 95% CI	Upper 95% CI
MARS-Fa total score-subjective quality	0.90	0.86	0.93
Subjective quality-engagement	0.85	0.77	0.90
Subjective quality-functionality	0.57	0.41	0.69
Subjective quality-aesthetics	0.79	0.73	0.85
Subjective quality-information quality	0.80	0.73	0.86
Engagement-functionality	0.38	0.20	0.53
Engagement-aesthetics	0.76	0.70	0.84
Engagement-information quality	0.75	0.66	0.82
Functionality-aesthetics	0.54	0.41	0.67
Functionality-information quality	0.52	0.35	0.68
Aesthetics-information quality	0.69	0.58	0.77

## Discussion

This is the first study that developed a translation and cultural adaptation of the MARS scale into the Farsi language. This study also included validation of the MARS-Fa tool with a sample of apps targeting smartphone addiction. The results show that the MARS-Fa is a reliable and valid tool that can be used to assess app quality. Health care professionals, researchers, authorities, organizations, and app developers can use this tool when developing new or evaluating existing apps in Farsi.

### Translation and Cultural Adaptation

Translating IT terminology tends to be challenging, especially in unrelated contexts such as health care. The employment of experts in the translation process facilitates the adaptation of scales, as discussed in the Italian MARS validation study [34]. To translate the tool, we employed 2 experts in IT concepts; the concepts were subsequently translated and then back translated into English by a third bilingual translator, similar to the Arabic MARS validation study [37]. The original scale developer and another English expert were asked to check each version. In the process, we excluded item 19, “Evidence base,” as it was not applicable because no apps were used in randomized controlled studies, as done in previous studies [29,34,44]. Nevertheless, given the complex terminology of the scale, it is recommended to develop a dedicated training module for Farsi-speaking app reviewers, such as the one developed for the original MARS [29] and the German version of the tool [35]. The training module will likely improve the interrater reliability, test-retest reliability, and possibly the validity of the MARS-Fa, but this needs to be formally tested in future studies, possibly with a different set of apps.

### Reliability

The MARS-Fa showed a good degree of interrater reliability, with ICCs ranging from 0.60 to 0.85, with results that are aligned with the original study (ICCs=0.79) [29] and other similar validation studies, such as the Italian (0.96) [34], Spanish (0.96) [36], German (0.83) [35], Arabic (0.84) [37], French (0.89) [40], Japanese (0.70) [38], and Turkish (0.94) [41] studies. Functionality was the domain with the lowest ICC value, as in the original MARS study (0.50) [29] and the Japanese study (0.40) [38]. This might be due to the nature of mHealth apps for mental health used in both studies, similar to the ones targeting smartphone addiction in this paper. It can also be due to differences in how raters interpreted the items. Training raters before using the instrument will likely reduce the likelihood of misinterpretations, as in the German study [35].

The MARS-Fa displayed a good internal consistency, with Cronbach α coefficients of both raters deemed “good” for the MARS total score. The Spearman-Brown split-half reliability indicated good internal consistency among the raters, as reported in the Italian MARS validation study [34]. Altogether, the internal consistency estimates of the MARS-Fa are aligned with similar MARS validation studies [34,36,37,40,41]. The functionality domain had a relatively lower level of internal consistency, as reported in the original MARS study (0.80) [29], and in other MARS-validation studies such as the Italian (0.82 between 2 raters) [34], Arabic (0.72) [37], French (0.79) [40], and Turkish (0.78 between 2 raters) [41] studies. A relatively low level of internal consistency estimates for the information quality subscale was also reported in other validation studies, such as the Italian (0.72 between 2 raters) [34], German (0.72) [35], and French (0.61) [40]. These differences might be due to the diverse nature of the tested apps, as functionality, navigation features, ease of use, and information included in each app can vary significantly between apps, depending on the type of health issue addressed and within apps, because content and format can vary across platforms and devices, as reported in Bardus et al [31].

Additionally, the MARS-Fa showed excellent test-retest reliability, as testified by significant and high Pearson correlations over time; all subscales and the total score were more than or equal to 0.90, according to methodology literature [45], indicating an excellent test-retest reliability.

### Validity

Overall, the MARS-Fa shows good construct validity, as all items seemed to correlate well within each objective subdomain. Similar to the Japanese validation study [38], one item of the functionality domain appeared to have the lowest correlation with the other items, “ease of use,“ which might indicate a wide variability in the usability of the apps analyzed. As for concurrent validity, the MARS-Fa total score (objective quality) was significantly correlated with subjective quality. However, this might be interpreted with caution as the subjective quality might be influenced by the reviewers’ completing the objective quality evaluation in the same instance, as discussed in the original MARS study [29] and reported in the Japanese validation study [38]. In the absence of other benchmarks, the correlation between the MARS-Fa total score and its subjective counterpart indicates that the two measures are somehow aligned.

### Strengths and Limitations

This is the first study reporting on the translation and cultural adaptation of the MARS scale into Farsi and its subsequent validation. A major strength of this study is the systematic process followed in translating and validating the MARS-Fa tool, using well-established and sound methodologies. The translation and cultural adaptation process involved IT and health sciences experts, who checked the content for and provided face validity. Furthermore, the construct and scale validation process followed a robust approach. The study involved 2 raters who independently assessed a systematically selected sample of apps. Through this process, the MARS-Fa tool can be reliably used to evaluate the quality of health apps in the Farsi language.

Limitations of this study include the fact that we tested the tool with a selected sample of apps for smartphone addiction. While the tool is intended to assess health apps in any domain, there might be some variability in the type of apps analyzed. Hence, we suggest that future studies test the MARS-Fa using other health apps. One of the limitations is that the MARS-Fa total score and objective subscales were validated against the subjective quality in the absence of an equivalent app quality evaluation scale. Future studies could compare the MARS-Fa to other app quality evaluation tools identified in the literature [24] to ascertain concurrent validity.

### Conclusions

The Farsi version of the MARS tool (MARS-Fa) is a reliable and valid instrument to assess mobile health app quality, as demonstrated by a sample of apps targeting smartphone addiction. Health experts, researchers, and app developers can use the MARS-Fa, to evaluate their apps or to assess groups of apps of the same kind. It can be easily accessed (Multimedia Appendix 1) free of charge. We hope that the MARS-Fa could be used as a criterion for evaluating apps before these are prescribed to patients.

## Appendix

Multimedia Appendix 1 List of mobile apps tested.

Multimedia Appendix 2 The Mobile App Rating Scale in Farsi (MARS-Fa).

## Abbreviations

ICC: intraclass correlation coefficient
MARS: Mobile Application Rating Scale
MARS-Fa: Mobile Application Rating Scale in Farsi
mHealth: mobile health

## Data Availability

The list of the tested mobile apps with the respective ratings is available in Multimedia Appendix 2. The Mobile App Rating Scale in Farsi (MARS-Fa) is available in Multimedia Appendix 1.

The data sets generated or analyzed during this study are available from the corresponding author upon reasonable request.
