# Supplementary material for: The Persian Version of the Mobile Application Rating Scale (MARS-Fa): Translation and Validation Study
Source: JMIR Form Res. 2022 Dec 5;6(12):e42225. doi: 10.2196/42225 (PMC9764158; doi:10.2196/42225)
Supplement: Multimedia Appendix 1 [file formative_v6i12e42225_app1.docx]

**دستورالعمل استفاده**

ارزیاب ها باید:

1. از برنامه استفاده کرده و حداقل 10 دقیقه آن را تست نمایند
2. (امتیاز هر بخش را) تعیین کنند
3. تنظیمات برنامه، اطلاعات برنامه نویس، پیوندهای خارجی، ویژگی های امنیتی و... را بررسی کنند.

**امتیازدهی**

**بخش**

A: میانگین امتیاز درگیرسازی:

B: میانگین امتیاز عملکرد:

C: میانگین امتیاز زیبایی:

D: میانگین امتیاز اطلاعات:

سوالات پاسخ داده شده با N/A را در امتیازدهی در نظر نگیرید.

میانگین امتیاز کیفیت برنامه: (A + B + C + D) / 4

E: میانگین امتیاز کیفیت ذهنی برنامه

F: میانگین امتیاز تاثیرِ مشاهده شده (اثربخشی برنامه بر رفتار کاربر)

مقیاس "کیفیت ذهنی برنامه" بسته به اهداف پژوهش می تواند به عنوان موارد منفرد یا به عنوان یک نمره متوسط گزارش شود.

مورد "تأثیر مشاهده شده" را می توان برای دستیابی به اطلاعات بر محور تأثیر برنامه در دانش، رفتارها و مقاصد مربوط به رفتار بهداشتی کاربر (هدف) تنظیم و استفاده کرد.

**شاخص رتبه بندی اپلیکیشن های موبایل (MARS):**

این بخش برای جمع آوری اطلاعات توصیفی و فنی اپلیکیشن ها می باشد. برای بدست آوردن اطلاعت زیر، بخش توضیحات Google play و iTunes را مطالعه بفرمایید:

نام اپلیکیشن:

رتبه این نسخه: رتبه تمامی نسخه ها:

توسعه دهنده (سازنده):

تعداد افرادی که این نسخه را رتبه دهی کرده اند: تعداد افرادی که کل نسخه ها را رتبه دهی کرده اند:

نسخه: آخرین آپدیت:

قیمت نسخه پایه: قیمت نسخه ارتقا یافته:

پلتفرم: iPhone iPad Android

توضیحات مختصر:

**تمرکز:** هدف اپلیکیشن (تمام گزینه هایی که جواب می باشند را انتخاب کنید )

افزایش احساس خوشی/شادی ذهن آگاهی/مدیتیشن/آرامش دهی

کاهش احساسات منفی افسردگی

اضطراب/ استرس عصبانیت

تغییر رفتار مصرف الکل یا مواد مخدر

هدف گزاری تفریح

ارتباطات سلامت فیزیکی

سایر

**استراتژی**: (تمام گزینه هایی که جواب می باشند را انتخاب کنید )

ارزیابی بازخورد

اطلاع رسانی/آموزش نظارت/پیگیری

هدف گزاری راهنمایی/مشاوره/استراتژی/آموزش مهارت

درمان شناختی رفتاری- رفتاری (اتفاقات مثبت)

درمان شناختی رفتاری- شناختی (به چالش کشیدن ذهن)

درمان بر اساس قبول کردن تعهد - ACT

ذهن آگاهی/مدیتیشن آرامش و تمدد اعصاب

سپاسگزاری و حق شناسی نقاط قوت سایر

**وابستگی نرم افزار به سازمان:**

نامعلوم تجاری دولتی NGO دانشگاه

**مناسبت سنی:** (تمام گزینه هایی که جواب می باشند را انتخاب کنید )

کودکان (زیر 12 سال) نوجوانان (12-17 سال)

جوانان (18-25 سال) بزرگسالان

عمومی

**ابعاد فنی نرم فزار:** (تمام گزینه هایی که جواب می باشند را انتخاب کنید )

قابلیت اشتراک گذاری (روی Facebook یا twitter و...)

اپلیکیشن دارای انجمن است

قابلیت محافظت با پسورد را دارد

لاگین (ورود به سیستم) نیاز دارد

یادآور ارسال می کند

برای عمل کردن نیاز به دسترسی به اینترنت دارد

**رتبه بندی کیفیت اپلیکیشن**

شاخص رتبه بندی، کیفیت اپلیکیشن ها را از چهار بعد ارزیابی می کند. تمامی سوالات در مقیاس لیکرت 5 تایی از: ((1 – ناکافی)) تا ((5 – عالی )) قرار دارند. در ارزیابی کیفی هر بعد، دور عددی که با دقت بیشتری کیفیت قسمت اپ را نشان می دهد خط بکشید. لطفا از توضیحات ابتدای هر بعد برای پاسخ دهی به سوالات استفاده کنید.

**بعد A**

**سرگرم شدن-** تفریح، جذابیت، قابلیت شخصی سازی، تعاملی بودن (به عنوان مثال فرستادن هشدار، پیام، یادآرور، بازخورد، قابلیت اشتراک گذاری)، تناسب با جامعه هدف.

**1. درگیرسازی: آیا اپلیکیشن برای استفاده مفرح و سرگرم کننده است ؟ آیا برای ساده سازی فعالیت های پیچیده از طریق بازی و سرگرمی برنامه ای دارد ؟**

1. اصلا سرگرم کننده نیست و کسل کننده می باشد.
2. اغلب خسته کننده است.
3. به اندازه ای سرگرم کننده و لذت بخش است که کاربر را برای مدت کوتاهی سرگرم کند. (کمتر از 5 دقیقه )
4. نسبتا سرگرم کننده و لذت بخش در حدی که کاربر را برای مدت زمانی 5 تا 10 دقیقه سرگرم می کند.
5. بسیار سرگرم کننده و لذت بخش است و فرد را برای استفاده مکرر تحریک می کند.

**2. جذابیت: آیا اپلیکیشن برای استفاده جذاب است؟ آیا راهکاری برای افزایش همکاری کاربران از طریق ارائه محتوا به شکل جذاب دارد؟**

1. به هیچ وجه جذاب نیست.
2. اغلب جذاب نیست.
3. نه جذاب است و نه غیر جذاب. کاربر را برای مدت کوتاهی جذب می کند. (کمتر از 5 دقیقه)
4. به اندازه متوسط جذاب است. برای مدت زمانی کاربر را جذب می کند ( 5–10 دقیقه)
5. بسیار جذاب است و کاربر را برای استفاده مکرر جذب می کند.

**3. شخصی سازی: آیا تمامی تنظیمات لازم برای قابلیت های اپ (مثل نوتیف و صدا و محتوا و...) را فراهم می کند؟؟**

1. اجازه شخصی سازی نمی دهد/ باید در هر بار استفاده، تنظیمات را وارد کرد.
2. اجازه شخصی سازی ناکافی آنهم برای عملکردهای محدودی را می دهد.
3. اجازه شخصی سازی پایه برای عملکرد به جا برنامه را می دهد.
4. اجازه شخصی سازی تنظیمات زیادی را می دهد.
5. اجازه شخصی سازی می تواند به تناسب خصوصیات و اولویت های فرد تنظیم و حفظ شوند.

**4. تعاملی بودن: آیا به کاربر اجازه وارد کردن اطلاعات میدهد؟ بازخورد دارد؟ و حاوی پیشنهاداتی در قالب یادآور ها، قابلیت اشتراک گذاری، اطلاع و.... می باشد؟**

**نکته: این عملکردها باید قابل شخصی سازی بوده و تعدادشان بسیار زیاد نباشد تا عالی باشند.**

1. بدون ویژگی های تعاملی و یا بدون پاسخ به تعاملات کاربر
2. ناکافی بودن تعامل، یا بازخورد، یا گزینه های ورود اطلاعات توسط کاربر که منجر به محدودیت قابلیت ها می شود.
3. ویژگیهای تعاملی پایه که منجر به عملکرد کافی نرم افزار می شود.
4. تعداد زیادی ویژگیهای تعاملی اعم از بازخورد، قابلیت ورود اطلاعات توسط کاربر و.. ارائه می دهد.
5. سطح بسیار بالایی از تعامل از طریق قابلیت های تعاملی، بازخورد و و ورود اطلاعات توسط کاربر فراهم شده است.

**5. گروه هدف:** **آیا محتوای نرم افزار (اطلاعات بصری، زبان، طراحی ) برای گروه هدف مناسب است؟**

1. کاملا نامناسب/نامفهوم/گیج کننده است.
2. اغلب نامناسب/نامفهوم/گیج کننده است.
3. قابل قبول است ولی هدفمند نیست. ممکن است نامناسب/نامفهوم/گیج کننده باشد.
4. هدفمند و متناسب با گروه هدف، همراه با مشکلات جزیی
5. کاملا هدفمند و متناسب با گروه هدف. مشکلی یافت نشد.

**میانگین امتیاز درگیرسازی: ______________**

**بعد B**

**کارایی** – کارایی اپلیکیشن، راحت بودن یادگیری، هدایت، منطق جریان عملکرد، طراحی نوع رفتار اپلیکیشن.

**6. کارایی: ویژگیها (عملکردها) و اجزای (دکمه ها/منوها) چقدر سریع/دقیق عمل می کنند؟**

1. اپلیکیشن ناکارآمد است. پاسخ دهی ناکارآمد/ ناکافی (مثل کرش/باگ/ویژگیهای ناکارآمد و..)
2. بعضی عملکردها کار می کنند. ولی کند هستند و یا مشکلات فنی اساسی دارند.
3. اپلیکیشن در کل کار می کند. بعضی مشکلات فنی نیاز به اصلاح دارند یا برنامه گاهی اوقات کند است.
4. اکثرا کار می کند ولی همراه با مشکلات کوچک یا بسیار جزئی.
5. پاسخ دهی عالی/به موقع. هیچ باگ فنی یافت نشد/دارای نشانگر برای مشخص کردن میزان زمان باقیمانده برای انجام عملیات می باشد.

**7. سهولت استفاده: یادگیری کار با اپلیکیشن چقدر آسان است؟ برچسب منو ها/آیکونها و دستور العمل ها چقدر واضح هستند؟**

1. بدون دستور المعمل یا با دستورالعمل محدود. برچسب منو ها/ آیکون ها گیج کننده و پیچیده هستند.
2. بعد از صرف زمان یا تلاش زیاد، قابل استفاده است.
3. بعد از صرف زمان یا تلاش متوسط قابل استفاده است.
4. یادگیری کار با اپلیکیشن آسان است یا دستورالعمل واضحی دارد.
5. به محض گرفتن نرم افزار می توان از آن استفاده کرد؛ بصری؛ ساده

**8. هدایت:** **آیا حرکت بین صفحات منطقی/دقیق/مناسب/بدون وقفه است؟ آیا لینک تمام صفحات ضروری وجود دارد؟**

1. بخش های مختلف نرم افزار از نظر منطقی به هم وصل نیستند/تصادفی/گیج کننده به نظر می آیند. گردش بین صفحات دشوار است.
2. قابل استفاده بعد از صرف زمان یا تلاش زیاد
3. قابل استفاده بعد از صرف زمان یا تلاش متوسط
4. استفاده آسان است یا فاقد لینک های جزئی می باشد.
5. کاملا منطقی، ساده، واضح و یا همراه با کلید های میانبر

**9. طراحی نوع رفتار:** **آیا تعاملات (ضربه زدن، کشیدن، پینچ"تغییر سایز یک آبجکت با کلیک همزمان بوسیله دو انگشت"، پیمایش) در تمام بخش ها و صفحات ثابت هستند؟**

1. کاملا بدون ثبات/ گیج کننده
2. غالبا بدون ثبات/گیج کننده
3. خوب است، به همراه مقداری بی ثباتی/گیج کنندگی
4. اکثرا باثبات و واضح است به همراه مشکلات جزئی
5. کاملا باثبات و واضح است.

**میانگین امتیاز کارایی: _______________**

**بعد C**

**زیبایی** – طراحی گرافیکی، جذابیت ظاهری کلی، طرح رنگ، ثبات استایل

**10. طرح یا قالب بندی: آیا ترتیب و اندازه دکمه ها/آیکونها/منو ها/محتوای صفحه مناسب و در صورت نیاز قابل بزرگنمایی است؟**

1. طراحی بسیار بد، به هم ریخته، بعضی از گزینه ها غیر قابل انتخاب/پیدا کردن/خواندن/دیدن هستند.ظاهر صفحات بهینه نیست.
2. طراحی بد، تصادفی، نامفهوم، بعضی از گزینه ها به سختی قابل انتخاب/خواندن/دیدن/پیدا کردن هستند.
3. رضایت بخش، مشکلات اندک با انتخاب/دیدن/پیدا کردن آیتمها و یا مشکل اندک در اندازه صفحه
4. اکثرا واضح، قابل انتخاب/دیدن/خواندن/پیدا کردن
5. حرفه ای، ساده، واضح، مرتب، سازماندهی منطقی، صفحه نمایش تنظیم و بهینه شده است. دلیل طراحی هر بخش مشخص است.

**11. گرافیک: گرافیک/رزولوشن استفاده شده در دکمه ها/نماد ها/منو ها/محتوا به چه میزان بالا است؟**

1. گرافیک غیر حرفه ای به نظر می آید، طراحی بصری بسیار ضعیف، بدون تناسب و ثبات در سبک
2. طراحی با کیفیت یا رزولوشن پایین، کیفیت پایین طراحی ظاهر. بدون تناسب و ثبات در سبک
3. کیفیت طراحی و طراحی گرافیکی متوسط می باشد (عموما سبک ثابت دارد)
4. طراحی ظاهر و گرافیک با کیفیت یا رزولوشن بالا می باشد. اکثرا متناسب و همراه با ثبات سبک
5. طراحی ظاهر و گرافیک با کیفیت/رزولوشن بسیار بالا می باشد. متناسب و در تمامی بخش ها همراه با ثبات

**12. جذابیت ظاهری: اپلیکیشن به چه میزان خوب به نظر میاد؟**

1. بدون ظاهر جذاب، نگاه کردن به آن ناخوشایند است. طراحی ضعیف، استفاده از رنگ های ناهماهنگ و متضاد.
2. با مقدار کمی جدابیت ظاهری، طراحی ضعیف، استفاده از رنگ های نامناسب، از نظر ظاهری خسته کننده
3. با مقداری جدابیت ظاهری، متوسط؛ نه خوشایند و نه ناخوشایند
4. سطح بالایی از جذایت ظاهری، گرافیک یکپارچه، طراحی باثبات و حرفه ای
5. همانند بالا **+** بسیار جذاب، خاطره انگیز، دور از انتظار، استفاده از رنگ ها سبب بهبود ویژگیها ها و منو های نرم افزار شده است.

**میانگین امتیاز ظاهر : ________________**

**بعد D**

**اطلاعات** – حاوی اطلاعات با کیفیت بالا (نظیر متن، بازخورد، اندازه گیریها، منابع) از منابع معتبر است. اگر موضوع نسبت به نرم افزار بی ربط بود N/A انتخاب کنید.

**13. دقت توضیحات نرم افزار(در اپ استور): آیا نرم افزار حاوی موارد توضیح داده شده است؟**

1. گمراه کننده. اپلیکیشن حاوی ویژگیها/قابلیت های ذکر شده در توضیحات نیست و یا توضیحات ندارد.
2. نادرست. نرم افزار حاوی مقدار کمی از ویژگیها/قابلیت های ذکر شده است.
3. خوب است. نرم افزار تعدادی از ویژگیها/قابلیت های ذکر شده را دارد.
4. صحیح. نرم افزار اکثر ویژگیها/قابلیت های ذکر شده را دارد.
5. توضیحات بسیار صحیح نرم افزار در مورد ویژگیها/قابلیت های ذکر شده.

**14. اهداف: آیا نرم افزار اهداف مشخص، قابل اندازه گیری و قابل دستیابی دارد؟ (در بخش توضیحات اپ استور و یا در داخل خود نرم افزار توضیح داده شده باشد)**

N/A، اهداف را فهرست نکرده و یا اهداف نرم افزار ربطی به اهداف پژوهش ندارد (به عنوان مثال استفاده از یک بازی برای اهداف آموزشی).

1. اپ هیچ شانسی در دستیابی به اهداف ذکر شده اش ندارد.
2. توضیحات یکسری از اهداف را فهرست کرده است، ولی اپ شانس کمی در دستیابی به آن اهداف دارد.
3. خوب است. اپلیکیشن اهداف مشخصی دارد که دستیابی به آن ممکن است.
4. اپلیکیشن اهدافی دارد که به وضوح مشخص شده است که قابل اندازه گیری بوده و دستیابی به آن ممکن است.
5. اپلیکیشن اهداف مشخص و قابل اندازه گیری دارد، احتمال دستیابی به اهداف بسیار بالا می باشد.

**15. کیفیت اطلاعات: آیا محتوای اپلیکیشن، صحیح، به خوبی نوشته شده، مرتبط به هدف/عنوان آن می باشد؟**

N/A، در اپلیکیشن هیچ اطلاعاتی وجود ندارد.

1. نامرتبط/نامناسب/ناصحیح/ناسازگار می باشد.
2. ضعیف. به سختی مرتبط/مناسب/سازگار/ممکن است صحیح باشد.
3. متوسط. تقریبا مرتبط/مناسب/سازگار/صحیح به نظر می آید.
4. مرتبط/مناسب/سازگار/صحیح می باشد.
5. بسیار صحیح/مناسب/سازگار/مرتبط می باشد.

**16. کمیت اطلاعات: آیا اهداف نرم افزار پوشش داده شده است؟؟ و این که آیا جامع و در عین حال مختصر می باشد؟**

N/A در اپ هیچ اطلاعاتی وجود ندارد.

1. حداقل میزان، یا منکوب کننده.
2. ناکافی یا ممکن است منکوب کننده باشد.
3. خوب است ولی جامع یا مختصر نیست.
4. طیف وسیعی از اطلاعات را ارائه می کند. مقداری شکاف اطلاعاتی دارد یا اطلاعاتی با جزئیات غیر ضروری دارد یا لینکی به اطلاعات و منابع بیشتر ندارد.
5. جامع و مختصر، لینک به اطلاعات و منابع بیشتر هم دارد.

**17. اطلاعات بصری: آیا توضیحات بصری مرتبط با مفاهیم – مثل: نمودار ها/تصاویر و ویدیو ها – واضح، منطقی و صحیح است؟**

N/A اطلاعات بصری در اپلیکیشن وجود ندارد (به طور مثال، فقط شامل متن و صوت است)

1. کاملاً نامفهوم/گیج کننده/غلط و یا بخش های ضروری را ندارد.
2. اغلب نامفهوم/گیج کننده/غلط.
3. خوب است ولی بعضی اوقات نامفهوم/گیج کننده/غلط است.
4. اکثراً واضح/منطقی/صحیح است، همراه با بعضی مشکلات جزئی.
5. کاملاً واضح/منطقی/صحیح است.

**18. قابل اطمینان بودن: آیا اپلیکیشن منبعی معتبر دارد؟ (در داخل اپ استور و یا خود اپلیکیشن توضیح داده شده باشد)**

1. منبع شناسایی شد ولی قابل اطمینان بودن/درستی آن مورد سوال است. (به عنوان مثال یک کسب و کار تجاری ذی نفع)
2. به نظر می آید منبعی معتبر دارد ولی نمی توان تایید کرد.(به عنوان مثال منبع صفحه ی وب ندارد)
3. توسط یک NGO کوچک/موسسه (بیمارستان یا مرکز)/ همراه با تامین مالی یک سازمان تجاری تخصصی
4. توسط دولت و یا دانشگاه و یا همانند مورد قبل ولی در سطح وسیع تر
5. توسط دولت رقابتی ملی تولید شده است و یا تامین مالی تحقیقات.(به عنوان مثال انجمن تحقیق استرالیا یا NHRMC)

**19. مبتنی بر شواهد: آیا اپلیکیشن آزمایش شده است؟ باید با شواهد تایید شود (در مقالات علمی منتشر شده)**

N/A، اپلیکیشن آزمایش نشده است.

1. شواهد نشان دهنده این است که اپلیکیشن کار نمی کند.
2. اپلیکیشن آزمایش شده است (به عنوان مثال در بخش های مقبولیت، کاربرد، سطح رضایت )و در آزمایش های تصادفی کنترل شده (RCT) بعضی قسمت ها نتایج مثبت دریافت کرده اند و یا مدارک کم و متناقص وجود دارد.
3. اپلیکیشن آزمایش شده است (به عنوان مثال در بخش های مقبولیت، کاربرد، سطح رضایت ) و نتایج مثبت داشته است در تحقیقاتی که آزمایش تصادفی کنترل شده (RCT) نبودند و مدارک متناقصی وجود ندارد
4. اپلیکیشن آزمایش شده و خروجی آن در یک یا دو RCT بررسی شده و نتایج مثبتی داشته است.
5. اپلیکیشن آزمایش شده است و خروجی آن در بیش از 3 RCT با کیفیت بالا بررسی شده و نتایج مثبتی داشته است.

**میانگین امتیاز اطلاعات بصری: ____________________**

*سوالاتی که با گزینه NA جواب داده شده است را محاسبه نکنید.

**کیفیت ذهنی اپلیکیشن**

**بعد E**

**20. آیا شما این اپلیکیشن را به افرادی که ممکن است از آن نفع ببرند معرفی می کنید؟**

1. **به هیچ وجه –** نرم افزار را به هیچ کس توصیه نمی کنم
2. ممکن است به تعداد کمی از افراد توصیه کنم
3. **شاید –** به تعدادی از افراد توصیه می کنم
4. تعداد زیادی از افراد وجود دارند که این اپ را به آنها توصیه می کنم
5. **حتماً –** این نرم افزار را به همه توصیه می کنم

**21. اگر این اپلیکیشن** **به شما مربوط می شد، فکر می کنید چند بار از آن در 12 ماه آینده استفاده می کردید؟**

1. هیچ
2. 1-2
3. 3-10
4. 11-50
5. 50<

**22. آیا برای این اپلیکیشن هزینه پرداخت می کنید؟**

1. قطعا نه
2. .
3. .
4. .
5. قطعا بله

**23. به این اپلیکیشن چند ستاره می دهید؟**

1. ***-** یکی از بدترین اپ هایی بود که استفاده کردم
2. **
3. *** - متوسط
4. ****
5. *****- یکی از بهترین اپ هایی که استفاده کردم

**تاثیرِ مشاهده شده**

این آیتم ها می توانند برای ارزیابی و درک تاثیر این اپ بر روی دانش، رفتار و قصد به تغییر کاربر و همچنین احتمال وقوع ان تغییرات در سلامت رفتار فرد مورد هدف استفاده شود.

**بعد F**

**1. آگاهی: به احتمال زیاد این اپلیکیشن آگاهی را نسبت به اهمیت اطلاع رسانی (درباره موضوع هدف گذاری شده) بالا می برد؟**

به شدت مخالفم به شدت موافقم

1 2 3 4 5

**2. دانش: به احتمال زیاد این اپلیکیشن دانش/فهم را در مورد (موضوع هدف گذاری شده) بالا می برد؟**

به شدت مخالفم به شدت موافقم

1 2 3 4 5

**3. رفتار: به احتمال زیاد این اپلیکیشن رفتار را (در مورد موضوع هدف گذاری شده) رو به بهبود می برد؟**

به شدت مخالفم به شدت موافقم

1 2 3 4 5

**4. قصد تغییر: به احتمال زیاد این اپلیکیشن انگیزه فرد را به سمت هدف افزایش می دهد؟**

به شدت مخالفم به شدت موافقم

1 2 3 4 5

**5. جستجوی کمک: احتمالاً استفاده از این اپ باعث تشویق فرد به کمک گرفتن بیشتر شود؟**

به شدت مخالفم به شدت موافقم

1 2 3 4 5

**6. تغییر رفتار: ممکن است استفاده از این اپلیکیشن باعث کاهش/افزایش{موضوع هدف گزاری شده} شود؟**

به شدت مخالفم به شدت موافقم

1 2 3 4 5
