# Supplementary material for: The Persian Version of the Mobile Application Rating Scale (MARS-Fa): Translation and Validation Study
Source: JMIR Form Res. 2022 Dec 5;6(12):e42225. doi: 10.2196/42225 (PMC9764158; doi:10.2196/42225)
Supplement: Multimedia Appendix 2 [file formative_v6i12e42225_app2.docx]

| **ID** | **AppName** | **A-Engagement** | **B-Functionality** | **C-Aesthetics** | **D-Information Quality** | **MARS Total score** | **E-Subjective Quality** |
| --- | --- | --- | --- | --- | --- | --- | --- |
| 1 | Your hour - Phone addiction Tracker and controller | 4.40 | 4.75 | 4.83 | 4.25 | 4.56 | 4.63 |
| 2 | Social Fever - Stop Smartphone Addiction | 4.00 | 4.38 | 4.33 | 4.00 | 4.18 | 3.38 |
| 3 | Phone addiction Blocker | 1.70 | 4.13 | 2.67 | 3.00 | 2.87 | 1.10 |
| 4 | TimeOut - Control Your Smartphone Addiction | 3.20 | 3.63 | 3.67 | 2.80 | 3.32 | 2.88 |
| 5 | StayFree - Phone Usage tracker & Overuse Reminder | 3.50 | 4.13 | 4.17 | 3.75 | 3.89 | 3.50 |
| 6 | Reservi - smartphone Addiction Timer Lock | 1.90 | 3.75 | 3.17 | 3.17 | 3.00 | 2.00 |
| 7 | AntiSocial : Phone Addiction | 3.90 | 4.13 | 4.33 | 3.92 | 4.07 | 4.50 |
| 8 | BlackOut : stay Focused / Beat Phone addiction | 2.50 | 4.38 | 3.83 | 3.00 | 3.43 | 2.38 |
| 9 | MyAddictoMeter - Mobile Addiction tracker | 2.90 | 3.75 | 3.83 | 3.75 | 3.56 | 2.88 |
| 10 | LessPhone Launcher – tone down your phone use | 2.20 | 2.88 | 2.50 | 2.88 | 2.61 | 1.75 |
| 11 | Smartphone addiction and cellphones advices (NOMOPHOBIA) | 4.00 | 4.00 | 4.67 | 4.08 | 4.19 | 4.00 |
| 12 | Space -Break phone addiction , stay focused | 4.40 | 4.25 | 4.67 | 4.08 | 4.35 | 4.75 |
| 13 | Smart Detox - Preventing SmartPhone Addiction | 4.20 | 4.38 | 4.50 | 3.67 | 4.19 | 4.25 |
| 14 | Quality Time - My Digital Diet | 4.00 | 4.13 | 3.83 | 3.35 | 3.83 | 3.13 |
| 15 | MyQualityTime | 3.40 | 3.38 | 3.17 | 3.17 | 3.28 | 2.00 |
| 16 | Anti SmartPhone addiction | 2.50 | 3.25 | 3.50 | 2.65 | 2.98 | 1.50 |
| 17 | Usage Tracker - Phone Addiction & Tracking | 3.20 | 4.25 | 4.00 | 3.35 | 3.70 | 3.00 |
| 18 | Detox Procrastination Blocker : Digital Detox | 3.10 | 4.50 | 4.50 | 3.17 | 3.82 | 3.13 |
| 19 | Digitox : digital wellbieng - screen time | 4.20 | 4.63 | 4.50 | 3.67 | 4.25 | 4.75 |
| 20 | My Phone Time - App usage Tracking - focus enabler | 3.20 | 4.00 | 3.67 | 3.50 | 3.59 | 3.25 |
| 21 | Usage Time -App Usage Manager | 3.30 | 4.50 | 3.67 | 3.67 | 3.78 | 3.38 |
| 22 | Phone Usage : Screen time monitor | 3.30 | 2.25 | 3.33 | 3.00 | 2.97 | 2.50 |
| 23 | Stay Focused - App Block | 3.40 | 4.25 | 3.83 | 3.50 | 3.75 | 3.25 |
| 24 | Addicted - Phone Usage Tracker | 1.80 | 4.00 | 2.83 | 2.98 | 2.90 | 1.85 |
| 25 | App Usage - Manage / Track Usage | 3.80 | 3.88 | 3.83 | 3.88 | 3.85 | 3.50 |
| 26 | ZenScreen - Track And limit screen time | 4.40 | 3.88 | 4.33 | 3.40 | 4.00 | 4.63 |
| 27 | Monitro - The App Usage Monitor | 2.10 | 3.63 | 3.00 | 2.80 | 2.88 | 1.38 |
| 28 | Mobile Addiction tracker - Anti Social | 3.50 | 4.38 | 4.00 | 3.75 | 3.91 | 3.38 |
| 29 | Sleepy - Addiction Remover | 2.00 | 4.13 | 3.17 | 2.83 | 3.03 | 1.88 |
| 30 | Focus Launcher - Minimal Launcher To stay Focused | 3.20 | 3.63 | 3.00 | 3.00 | 3.21 | 3.38 |
| 31 | AddiLock : Lock Your Phone Addiction | 1.70 | 3.50 | 3.17 | 2.50 | 2.72 | 1.50 |
| 32 | Mobile Addiction Meter Pro | 2.20 | 4.00 | 2.50 | 1.75 | 2.61 | 1.75 |
| 33 | Phone Usage And Addiction Tracker | 1.70 | 4.13 | 2.83 | 3.10 | 2.94 | 2.25 |
| 34 | Keep Me Out | 2.50 | 4.25 | 3.67 | 3.25 | 3.42 | 2.63 |
| 35 | App Off Timer | 2.90 | 4.13 | 4.00 | 3.20 | 3.56 | 2.88 |
| 36 | The Perfect Day | 1.70 | 2.25 | 2.33 | 1.40 | 1.92 | 1.00 |
| 37 | Minify - Restrict your smartphone Usage | 2.80 | 3.88 | 2.67 | 3.15 | 3.12 | 2.25 |
| 38 | FOMO - Beat Your Phone Addiction | 2.40 | 4.13 | 4.00 | 2.73 | 3.31 | 3.13 |
| 39 | Phone Usage Tracker : Phone Usage manager | 2.90 | 3.75 | 3.33 | 2.93 | 3.23 | 2.38 |
| 40 | iFocusMode - App Block For No Phone Addiction | 3.40 | 3.38 | 3.17 | 3.42 | 3.34 | 3.38 |
| 41 | Bfree - Break Phone Addiction By Doing Math Problems | 3.90 | 3.25 | 3.50 | 2.67 | 3.33 | 1.98 |
| 42 | addictOMeter phone Manager | 2.30 | 2.75 | 3.33 | 3.25 | 2.91 | 1.75 |
| 43 | Unhook : Screen Time (Reduce your phone Usage) | 1.90 | 4.38 | 3.83 | 3.00 | 3.28 | 2.88 |
| 44 | Moment – Screen Time Tracker | 4.10 | 4.00 | 4.83 | 3.42 | 4.09 | 3.63 |
| 45 | lockMeOut- Stay Focused, Be Proactive: SaveMyTime | 2.30 | 3.63 | 3.33 | 2.60 | 2.96 | 2.50 |
| 46 | Tracky - A Digital Wellbeing Helper | 3.10 | 3.88 | 4.17 | 3.33 | 3.62 | 2.88 |
| 47 | ActionDash: Digital Wellbeing & Screen Time helper | 3.70 | 4.00 | 4.00 | 3.50 | 3.80 | 4.00 |
| 48 | RescueTime Time Management and Digital Wellness | 3.60 | 3.75 | 4.00 | 3.58 | 3.73 | 3.38 |
| 49 | Usage Analyzer: Apps, Data & History | 3.40 | 4.00 | 4.50 | 3.42 | 3.83 | 3.75 |
| 50 | Usage Time. Smartphonoholic | 2.30 | 4.38 | 3.83 | 3.42 | 3.48 | 2.75 |
| 51 | TimeGurus – Time Management , Usage tracker | 2.80 | 3.50 | 3.00 | 3.00 | 3.08 | 2.88 |
| 52 | Luna - Kid Launcher & Parental controls | 3.60 | 3.88 | 4.17 | 3.50 | 3.79 | 4.00 |
| 53 | Study & Play | 2.40 | 4.13 | 2.50 | 3.13 | 3.04 | 2.50 |
| 54 | Apna Time: Check Phone Usage and compete | 2.40 | 3.88 | 3.33 | 2.90 | 3.13 | 2.25 |
| 55 | HelpMeFocus - Block Distractions, Stay Focused. | 3.50 | 4.25 | 4.33 | 3.38 | 3.86 | 3.38 |
| 56 | Lokeet | 1.20 | 3.25 | 2.50 | 1.80 | 2.19 | 1.10 |
| 57 | Mobile Fence Parental Control | 4.00 | 4.00 | 4.17 | 4.17 | 4.08 | 4.50 |
| 58 | SELFLOCK - Lockscreen usage | 2.70 | 3.13 | 3.00 | 2.60 | 2.86 | 2.00 |
| 59 | BlockSite - Block Distracting Apps & Sites | 3.60 | 4.63 | 4.17 | 3.57 | 3.99 | 4.75 |
| 60 | Town Timer - be focused and keep track of time | 4.40 | 4.50 | 4.50 | 3.75 | 4.29 | 4.75 |
| 61 | Mobile Addiction Meter - Unlock Counter | 1.10 | 3.88 | 1.33 | 1.90 | 2.05 | 1.00 |
| 62 | App statistics: Track Usage, App Usage | 2.60 | 4.00 | 3.83 | 3.58 | 3.50 | 3.38 |
| 63 | Focus: Limit Distracting Apps, End Procrastination | 2.40 | 3.63 | 1.67 | 2.75 | 2.61 | 2.00 |
| 64 | Usage Tracker- Phone | 1.70 | 3.38 | 3.50 | 2.50 | 2.77 | 1.75 |
| 65 | AppTime Pro - phone usage tracker | 3.30 | 4.50 | 4.17 | 3.92 | 3.97 | 4.13 |
| 66 | B. Focused - Phone Blocker - Time to be focused | 2.90 | 4.38 | 4.33 | 3.20 | 3.70 | 3.75 |
| 67 | Flipd — Stay Focused, Remove Distractions | 3.70 | 4.25 | 5.00 | 3.25 | 4.05 | 4.50 |
| 68 | Phone Usage Time | 2.40 | 3.75 | 2.83 | 2.90 | 2.97 | 3.25 |
| 69 | RealizD - Track how much you use your phone | 4.00 | 4.38 | 4.50 | 4.00 | 4.22 | 3.88 |
| 70 | Moment - balance screen time | 4.10 | 4.13 | 4.83 | 3.42 | 4.12 | 3.63 |
| 71 | Scheck : Phone Addiction Quiz | 2.60 | 4.63 | 3.83 | 2.80 | 3.46 | 1.88 |
| 72 | Focus by Axon | 3.30 | 3.63 | 4.00 | 2.85 | 3.44 | 2.50 |
| 73 | SPACE - Break phone Addiction | 4.10 | 4.00 | 4.00 | 3.75 | 3.96 | 4.38 |
| 74 | Nobile Alarm | 2.30 | 3.75 | 3.17 | 3.00 | 3.05 | 3.25 |
| 75 | BreakFree - track Screen Time! | 2.90 | 3.63 | 3.67 | 3.00 | 3.30 | 3.13 |
| 76 | BSOCIABLE - An Effective Way To Control Your Smartphone Addiction! | 3.30 | 3.38 | 3.50 | 2.67 | 3.21 | 2.63 |
| 77 | Samrtphone addiction and Usage | 3.80 | 4.00 | 4.33 | 3.75 | 3.97 | 3.75 |
| 78 | Mute - less screen time | 4.10 | 4.38 | 4.33 | 3.58 | 4.10 | 4.00 |
| 79 | habit time tracker and control | 3.80 | 4.13 | 3.67 | 3.67 | 3.81 | 3.88 |
| 80 | The MILK App | 3.70 | 4.25 | 4.33 | 3.42 | 3.93 | 4.13 |
| 81 | RealizD - Screen Time Tracker | 4.30 | 4.25 | 4.33 | 3.83 | 4.18 | 4.13 |
| 82 | Lightdogs : Focus Timer | 4.10 | 4.63 | 4.50 | 3.20 | 4.11 | 4.38 |
| 83 | Focus Drive - Pomodoro Timer | 3.00 | 4.00 | 3.83 | 2.75 | 3.40 | 2.50 |
| 84 | Pawductivity | 2.80 | 4.38 | 4.17 | 2.83 | 3.54 | 3.00 |
| 85 | SCRIIN. | 3.50 | 3.63 | 4.33 | 3.17 | 3.66 | 3.13 |
| 86 | FishCure : stay focused | 4.20 | 4.38 | 4.67 | 3.83 | 4.27 | 4.75 |
| 87 | Ubhind - Mobile Life Pattern | 3.20 | 3.63 | 3.83 | 3.58 | 3.56 | 3.25 |
| 88 | Checky - Phone Habit Tracker | 2.00 | 3.63 | 3.00 | 2.30 | 2.73 | 1.38 |
| 89 | Instant - Life log Screen time | 3.50 | 3.63 | 4.17 | 3.58 | 3.72 | 3.88 |
| 90 | (OFFTIME) light - Track how much you use your phone & digital detox and unplug to focus | 3.60 | 4.00 | 3.83 | 3.33 | 3.69 | 3.25 |
| 91 | Forest Grow - Motivation Alarm | 3.30 | 3.75 | 4.00 | 2.63 | 3.42 | 2.50 |
| 92 | ShutApp - Digital Detox | 3.20 | 4.38 | 4.50 | 3.00 | 3.77 | 3.63 |
